# Editorial: Women in Science - Ophthalmology 2021

**DOI:** 10.3389/fmed.2022.966392

**Published:** 2022-06-30

**Authors:** Menaka C. Thounaojam

**Affiliations:** ^1^Department of Ophthalmology, Medical College of Georgia, Augusta University, Augusta, GA, United States; ^2^James and Jean Culver Vision Discovery Institute, Medical College of Georgia, Augusta University, Augusta, GA, United States

**Keywords:** Ophthalmology, UNESCO, stem, public health, medicine

According to 2016 data from the UNESCO Institute for Statistics (UIS), women make up fewer than 30% of science, technology, engineering, and mathematics (STEM) researchers. Although, at the undergraduate level, the proportion of women and men studying STEM is roughly equal, women are underrepresented in top positions in medicine ^1^. Nevertheless, many extremely influential and accomplished women in the field of Ophthalmology are contributing to the field and solving important questions. On the other hand, female scientists continue to be underrepresented in various facets of academic life. Several initiatives to promote the visibility of women in science have lately been launched (e.g., awards for women in STEM). Under-representation, income disparities, and increased career-related problems continue to be obstacles for women in scientific disciplines. Despite decades of progress, the COVID-19 pandemic has exacerbated these issues, emphasizing the so-called “leaky pipeline,” a model that depicts how women in science, technology, engineering, and mathematics (STEM) have missed opportunities due to gender bias and structural barriers (1). The COVID-19 pandemic also widened the gender divide in academic publishing (2). Even though female academic participation in Ophthalmology has increased in past decades, recent findings demonstrate that COVID-19 has reversed this trend. During the pandemic, women's contributions to COVID-19 Ophthalmology scholarship were much lower than predicted, particularly in senior roles (2). This Research Topic is aimed to create the frame for promoting and recognizing the scientific achievements of women researchers in Medicine, specifically in the field of Ophthalmology. Herein, we discuss 11 accepted articles wherein either the first or corresponding author identifies as female, collectively representing multiple countries worldwide.

Consistent with the theme of this Research Topic, Cao et al. evaluated the extent of the gender gap in citations in Ophthalmology literature between August 2015 and July 2020. This study suggested that despite the increase in female-led papers from August 2015 to July 2020, the proportion was still found to be much lower than that of the male-led papers. With the recent worldwide SARS-CoV-2 pandemic, several papers developed novel diagnostic tools for COVID-19 based on either molecular or imaging data. Hohberger et al. used Optical coherence tomography angiography (OCT-A) to evaluate the changes in the retinal vasculature and proposed that OCT-A observations could be correlated with systemic microcirculation and serve as clinical markers of severity of COVID-19 disease.

This Research Topic also included two case reports on two genetic conditions, a congenital disorder of glycosylation-Ia and CAFDADD syndrome (Cardiac, facial, and digital anomalies with developmental delay). A case presented by Midena and Pilotto confirmed the essential role of multimodal retinal imaging, a non-invasive way to provide new insights into the retinal involvement of patients affected by CDG-Ia. Additionally, multimodal retinal imaging associated CDG-Ia with multiple, bilateral, calcific retinal astrocytic hamartomas. A case report by Paprocka et al. detected *de novo* variant c.1708C>G [p.(His570Asp)] in TRAF7 in two pediatric patients with the very rare CAFDADD syndrome. Their studies indicated that a TRAF7 mutation should be evaluated in patients with characteristic dysmorphic features, especially within the palpebral fissure (blepharophimosis and/or ptosis), congenital defects of the heart and skeleton, and psychomotor delay.

Two of the articles on the utility and proper clinical usage of pattern electroretinogram and *in vivo* confocal microscopy for better patient data interpretation were also included in this Research Topic. Firstly, a study by Friedel et al. evaluated potentially more feasible procedures for PERG recordings in daily diagnostics in psychiatry. The authors were able to methodologically improve the recording procedure by demonstrating the suitability of a higher stimulation frequency for recordings and introducing an interpersonal normalization approach for the PERG signals, further enhancing the sensitivity of the method. Next, a study by Zhang et al. reported that to minimize the likelihood of under-sampling at the participant level of a study, the average cell density value from quantifying 12 random, non-overlapping IVCM images (400 × 400 μm) should be used for corneal epithelial IC density estimates for the central cornea, and seven equivalent images should be used for the peripheral cornea instead of the traditional “three representative images” approach.

Three more clinical studies with significant findings for myopia, primary open-angle glaucoma, and diabetic retinopathy were published. In China, vitamin D insufficiency is far more common than in other countries. Li et al. conducted a study to see if vitamin D is linked to myopia in these two groups of participants, who had different vitamin D levels due to various diets and sun exposure. They discovered that total serum vitamin D content does not affect myopia growth in Chinese children and adolescents, which suggests that the links between outdoor exposure and myopia progression should be investigated further. Improved medication adherence in the days leading up to clinic visits is referred to as whitecoat adherence. Poleon et al. conducted research to assess and identify parameters linked to whitecoat adherence. Within 3 days of the clinic appointment, they noticed a considerable increase in adherence. According to the authors, patients with higher levels of adherence may also have higher levels of healthcare engagement, prompting them to focus more significantly on their adherence, particularly before the clinic appointment. Tear fluid biomarkers may provide a non-invasive technique for diagnosing diabetic patients at risk of developing diabetic retinopathy (DR), improving diagnostic accuracy and understanding of the disease's etiology. Amorim et al. looked at the tear fluid of non-diabetic individuals, diabetic patients with no DR, and diabetic patients with non-proliferative DR (NPDR) or proliferative DR (PDR) to see if there were any potential biomarkers for DR diagnosis and staging. They discovered alterations in many proteins in tear fluid linked to various biological processes related to DR, such as oxidative stress, immunological response, and inflammation. The authors also urged that their findings be validated in a more extensive study with a larger sample size and suggested that the identification of a set of biomarkers could improve the early diagnosis of DR and assure quick treatment for this vision-threatening disease.

Finally, two review pieces were published in this Research Topic. To begin, Adamus examined the role of immunological reactions in the course of retinal degeneration caused by gene mutations. Inherited retinal diseases (IRDs) are a group of clinically and genetically diverse uncommon disorders that cause retinal malfunction and photoreceptor cell death, resulting in blindness. Retinitis pigmentosa (RP), which affects 1 in every 4,000 people worldwide, is one of the most common and severe forms of these retinopathies. This review paper meticulously assembled pertinent research and offers insight into the present function of autoimmunity/immunity in RP pathogenesis. Ocriplasmin is a recombinant protease that can be injected into the vitreous cavity to treat this illness; however, there is still debate about its efficacy and safety, especially regarding patient selection. Chen et al. conducted a comprehensive analysis of articles published before August 2020 to determine the efficacy and safety of ocriplasmin treatment on symptomatic vitreomacular adhesion (sVMA). The authors concluded that ocriplasmin is a viable treatment option for sVMA based on evidence from five randomized controlled trials and 50 cohort studies.

While the pandemic has reformed the workspaces and increased challenges in female authorship, more efforts are required to bridge the gender gap. In this regard, this Research Topic is a small effort to acknowledge the contribution of excellent female scientists worldwide in the field of Ophthalmology.

## Author Contributions

The author confirms being the sole contributor of this work and has approved it for publication.

## Conflict of Interest

The author declares that the research was conducted in the absence of any commercial or financial relationships that could be construed as a potential conflict of interest.

## Publisher's Note

All claims expressed in this article are solely those of the authors and do not necessarily represent those of their affiliated organizations, or those of the publisher, the editors and the reviewers. Any product that may be evaluated in this article, or claim that may be made by its manufacturer, is not guaranteed or endorsed by the publisher.

## Footnotes

^1^ http://uis.unesco.org/en/topic/women-science
